# RhesusBase: a knowledgebase for the monkey research community

**DOI:** 10.1093/nar/gks835

**Published:** 2012-09-08

**Authors:** Shi-Jian Zhang, Chu-Jun Liu, Mingming Shi, Lei Kong, Jia-Yu Chen, Wei-Zhen Zhou, Xiaotong Zhu, Peng Yu, Jue Wang, Xinzhuang Yang, Ning Hou, Zhiqiang Ye, Rongli Zhang, Ruiping Xiao, Xiuqin Zhang, Chuan-Yun Li

**Affiliations:** ^1^Institute of Molecular Medicine, Peking University, Beijing, ^2^Center for Bioinformatics, National Laboratory of Protein Engineering and Plant Genetic Engineering, College of Life Sciences, Peking University, Beijing, and ^3^Drug Discovery Center, Key Laboratory of Chemical Genomics, Peking University Shenzhen Graduate School, Shenzhen, China

## Abstract

Although the rhesus macaque is a unique model for the translational study of human diseases, currently its use in biomedical research is still in its infant stage due to error-prone gene structures and limited annotations. Here, we present RhesusBase for the monkey research community (http://www.rhesusbase.org). We performed strand-specific RNA-Seq studies in 10 macaque tissues and generated 1.2 billion 90-bp paired-end reads, covering >97.4% of the putative exon in macaque transcripts annotated by Ensembl. We found that at least 28.7% of the macaque transcripts were previously mis-annotated, mainly due to incorrect exon–intron boundaries, incomplete untranslated regions (UTRs) and missed exons. Compared with the previous gene models, the revised transcripts show clearer sequence motifs near splicing junctions and the end of UTRs, as well as cleaner patterns of exon–intron distribution for expression tags and cross-species conservation scores. Strikingly, 1292 exon–intron boundary revisions between coding exons corrected the previously mis-annotated open reading frames. The revised gene models were experimentally verified in randomly selected cases. We further integrated functional genomics annotations from >60 categories of public and in-house resources and developed an online accessible database. User-friendly interfaces were developed to update, retrieve, visualize and download the RhesusBase meta-data, providing a ‘one-stop’ resource for the monkey research community.

## INTRODUCTION

As a non-human primate, the rhesus macaque has unique advantages in molecular and translational studies (1). On one hand, although rodents are widely used in molecular mechanism studies and drug preclinical evaluation, fundamental differences in genome sequence composition, expression regulations, pharmacokinetics and behavior have been demonstrated between human and these small-animal models (1). The extension of molecular mechanism from rodents to humans should be considered with care in regard to diseases and drug development (1). On the other hand, experimental models of human behaviors and diseases are limited, due to environmental factors such as differences in diet or drug use, that contribute substantially to their pathogenesis (2) and leading to controversial findings (3). Subsequent studies of mechanisms are also hampered by difficulties in patient sample collection. In contrast, the rhesus macaque has advantages as a central model animal (4). Especially, as a species closely related to human, the genome sequence composition and expression regulation are more similar (1,5), making it a unique model for studying the physiological and pathological features of disease, identifying the causal genetic relationships between genotypes and phenotypes, underpinning the molecular mechanisms underlying complex diseases, and assessing the effectiveness and side effects of new drugs.

Although the rhesus macaque has unique advantages, its current use in biomedical research is still limited, partly due to error-prone gene structures and limited functional genomics annotations. After the first declaration of the rhesus macaque genome in 2007 (1), functional genomics data started to accumulate, but the available annotations are still scarce. One example is the transcriptional expression data traditionally used in transcript structure definition: according to the latest statistics from the National Center for Biotechnology Information (6), only 60 267 Expressed Sequence Tags (ESTs) have been reported in rhesus macaque, two orders of magnitude fewer than in the human (Build 37.3, 8 315 296 ESTs). For the majority of genes in the rhesus macaque, the transcript structure thus mainly relies on *ab initio* or comparative genomics-guided predictions, with only ∼1% supported by real mRNA and EST data according to recent RefSeq statistics (7,8). The transcript structures in 28.7% of rhesus macaque genes have been mis-annotated by the current annotation system as demonstrated by the current study, posing a major challenge in the monkey research community.

Even the limited annotations for rhesus macaque are widely scattered in the literature or in specialized databases without systematic integration. One example is for single nucleotide polymorphism (SNP) data: although at least four databases, dbSNP (8), MamuSNP (9), MonkeySNP (10) and CMSNP (11), have been developed to integrate monkey genotyping data, a standardized data structure or quality control mechanism is still lacking to efficiently manage the meta-data generated by different methodologies. Another example is for monkey transcription expression profiles identified by next-generation sequencing technology (12). Although such studies have been carried out in multiple monkey tissues with limited tissue selections and sequencing depth (5,13–17), it is not straightforward for biologists to take full advantages of the RNA-Seq data on accurate expression quantification and *de novo* splicing structure definition (12). A comprehensive platform is thus urgently needed in the community to effectively integrate and visualize such high-throughput data.

Overall, it is important to study novel gene functions and disease mechanisms in the framework of a well-annotated genomic context, which can provide state-of-the-art insights from the perspective of comparative genomics, gene regulation, expression patterns and evolutionary clues. Currently, ‘FlyBase’ (18), ‘WormBase’ (19) and Mouse Genome Informatics (20) have been established, which greatly enhance the international study of fruit flies, nematodes and mice. Here, we present the first comprehensive ‘RhesusBase’ effort in the rhesus monkey, to refine genome-wide gene structures, to integrate >60 categories of public and in-house functional annotations, and to develop the first user-friendly knowledgebase platform, providing a ‘one-stop’ resource for the monkey research community.

## MATERIALS AND METHODS

### Ethics statement

Rhesus monkeys tissues were obtained from the Institute of Molecular Medicine in Peking University, which has an animal facility internationally accredited by the Association for Assessment and Accreditation of Laboratory Animal Care (AAALAC). This study was approved by the Institutional Animal Care and Use Committee of Peking University. All animals were handled in strict accordance with good animal practice as defined by the relevant national and local animal welfare bodies.

### Computational processing of strand-specific poly (A)-positive RNA-Seq data

Total RNA was extracted from 10 rhesus monkey tissues using the Trizol method and analysed by an Agilent 2100 bio-analyzer (Agilent Technologies). The strand-specific Poly (A)-positive RNA-Seq study was performed on 10 rhesus macaque tissues, with the Illumina HiSeq2000 platform running 90 cycles with paired-end design according to the manufacturer’s instructions. In-house paired-end mRNA sequence tags were mapped to the rhesus monkey genome (rheMac2) by BWA (v0.5.9) (21) and TopHat (v1.2.0) (22). Multiple alignment reads were discarded. A series of Perl (v5.12.2) and R (v2.13.1) scripts were implemented to process and evaluate the quality of the RNA-Seq data, and calculate the statistics of genes, transcripts, exons and splicing junctions (Table 1).

**Table 1.** Statistics of RNA-Seq coverage on fine-scale monkey transcript structure

Categories	Total^a^	Covered^b^	Percentage
Exons	360 789	351 311	97.4
Junctions	317 969	273 967	86.2
Transcripts	42 820	33 914	79.2

^a^Number of exons, junctions or transcripts on the basis of Ensembl gene models.

^b^Number of exons, junctions or transcripts covered by expression tags.

### Genome-wide refinement of monkey gene structures

The fine-scale structures in monkey transcripts were revised on the basis of the RNA-Seq data. First, an exon/intron boundary was revised when (i) the new splicing model was supported by at least two expression tags across the splicing junction, while no tag supporting the previous splicing model; (ii) the expression tags supporting both the donor and acceptor sites and the splicing junctions were marked with GT–AG, GC–AG and AT–AC (23); and (iii) the revised splicing junction was located within the start site of the leading exon and the end site of the followed exon, creating revised exons with no shorter than 80% and no longer than 120% of the length for previously defined exons by Ensembl. Second, on the basis of the distribution of mRNA expression tags on the genome, we extended the 5′- and 3′-UTRs of the previous gene model to a new stop site, where (i) the base-level coverage of the expression tags was <15 in at least two samples; and (ii) when combining its upstream sites with identical tag coverage and the following sites with identical tag coverage, the average base-level coverage of the expression tags is <15 in each sample. Revisions with <100-bp extension were not included. Third, we identified potential new exons missed by the current annotation using Cufflink (v 0.9.3) with parameters -o -F 0.4 -j 0.45 -m 220 -p 4 (24). An exon was defined when (i) it was supported by continuous expression tags and defined by Cufflink as an intact exon; (ii) it was located in a previously annotated transcript; (iii) for both ends of the new exon, at least two expression tags linked it to known gene model; and (iv) the overlap between the new exon and all other annotated exons was <30%. Finally, we also identified 8057 brand-new transcripts using similar approach. A new transcript had at least two intact exons connected by splicing junctions, supported by at least two expression tags. Moreover, the whole transcript was located in intergenic regions as defined by the current Ensembl annotation. New transcripts were clustered following the Genome-based UniGene Build Procedure (6). A series of Perl (v5.12.2) scripts were implemented to refine the fine-scale transcript structures (Table 2; Supplementary Figures S1–S3 and Supplementary Table S1).

**Table 2.** 28.7% Ensembl macaque transcripts were convincingly refined

Categories	Events	Transcripts	Percentage^a^
Junctions	4 054	2 947	6.9
5′UTRs	2 267	2 267	5.3
3′UTRs	7 917	7 917	18.5
New exons	2 427	1 602	3.7
Total	16 665	12 303^b^	28.7

^a^Percentage of revised Ensembl transcripts.

^b^Number of transcripts involved in four types of refinements. Transcripts with two or more revisions were counted once.

### Evaluation of transcript structure refinement

**Figure 1.**
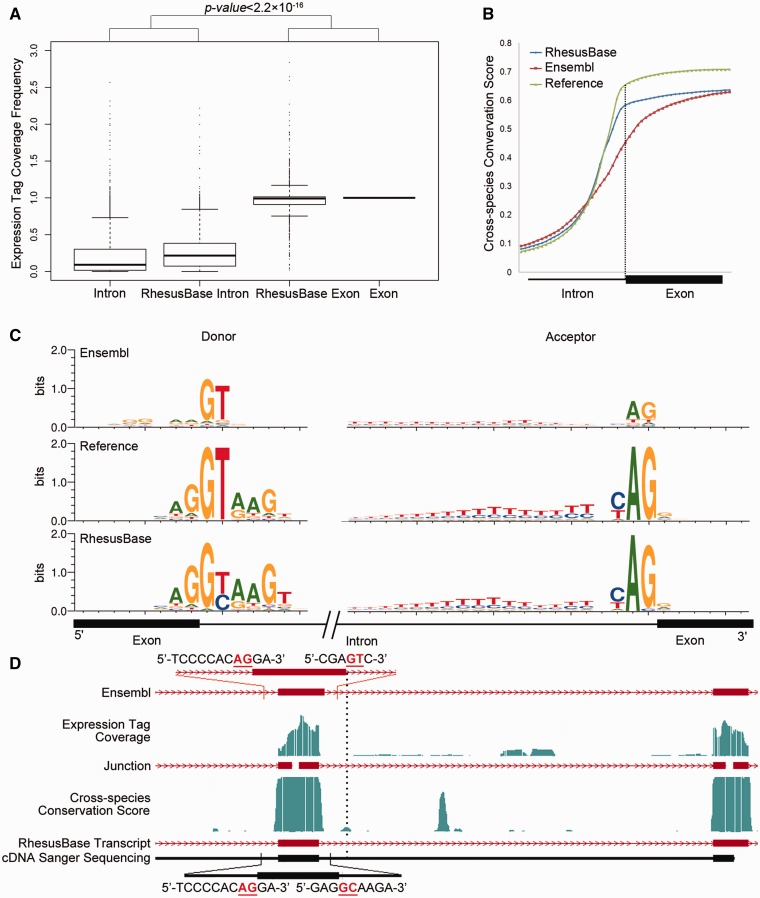
Evaluation of refined exon/intron boundaries. (**A**) Normalized mRNA-Seq expression tag coverage for each refined splicing junction in different categories. Exon: exonic regions defined by both gene models; Intron: intronic regions defined by both gene models; RhesusBase Exon: exonic regions defined by revised gene models, while intronic regions by previous gene models; RhesusBase Intron: intronic regions defined by revised gene models, while exonic regions by previous gene models; (**B**) Intron-exon distributions of cross-species conservation score. Reference: splicing junction supported by both gene models; Ensembl: splicing junction defined by Ensembl; RhesusBase: refined splicing junction in this study. (**C**) Sequence motifs flanking the splicing junctions calculated on the basis of previous gene models (Ensembl) and revised gene models (RhesusBase). Reference: distribution calculated using 242 603 splicing junctions supported by both gene models with at least two independent expression tags across the splicing junction; Ensembl/RhesusBase: distributions calculated using 1793 acceptor sites and 2261 donor sites on the basis of previous gene models and revised gene models. (**D**) One example of a revised transcript. Both the previous gene models (Ensembl) and the revised gene models (RhesusBase) are shown. RNA-Seq expression tag coverage and splicing junctions indicated by expression tags across junctions, cross-species conservation score, as well as sequenced cDNA fragments are aligned accordingly. Strand information is indicated by arrows on transcripts and exon boundaries are indicated by vertical dashed lines. The sequence surrounding the splicing junction is indicated, in which GT–AG or GC–AG sites are highlighted in red.

**Figure 2.**
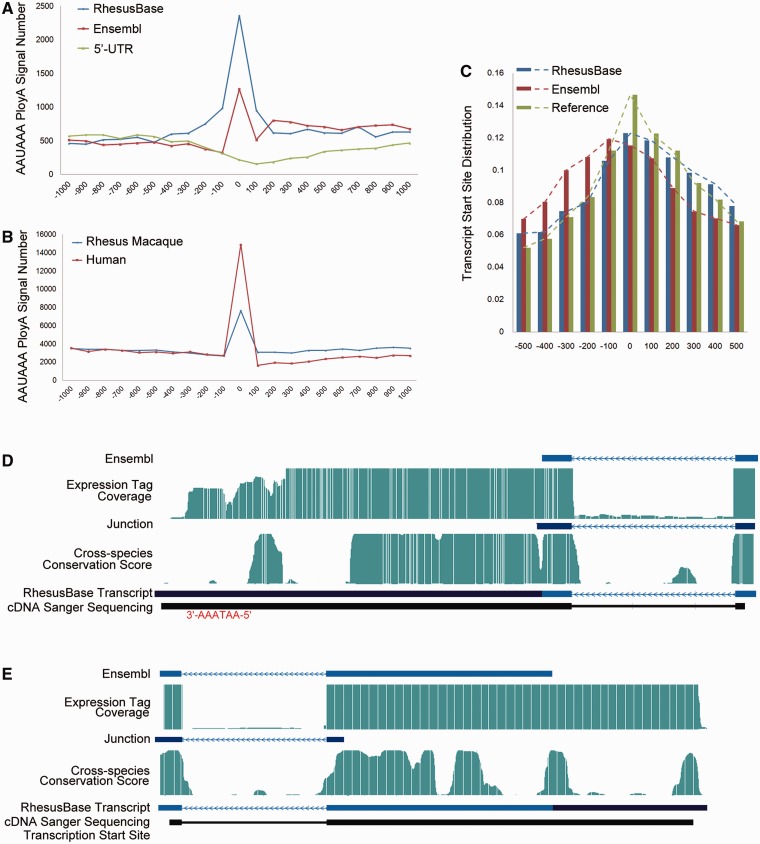
Evaluation of extended 5′- or 3′-UTRs. (**A**) Frequencies of AAUAAA hexamer near the end of the 3′-UTRs, on the basis of previous gene models (Ensembl) and the revised gene models (RhesusBase). Negative controls were generated using flanking regions near the start site of these transcripts (Negative Controls). (**B**) Frequencies of AAUAAA hexamer near the end of the 3′-UTRs, for transcript annotations in human and Ensembl annotations in rhesus macaque. (**C**) Distribution of the transcription start sites identified by ChIP-Seq study, on the basis of the previous and revised gene models. Reference: the end of the 5′-UTR supported by both previous and new models; (D and E) Gene structures of two experimentally verified transcripts revised by RhesusBase. Both the previous gene models (Ensembl) and the revised gene models (RhesusBase) are shown. RNA-Seq expression tag coverage, splicing junctions, cross-species conservation score, as well as sequenced cDNA fragments were aligned accordingly. AATAAA site (**D**) or transcription start site (**E**) identified by ChIP-Seq study are highlighted. The RNA-Seq expression tag coverage was set to the maximal score for sites with high tag coverage (>100).

**Figure 3.**
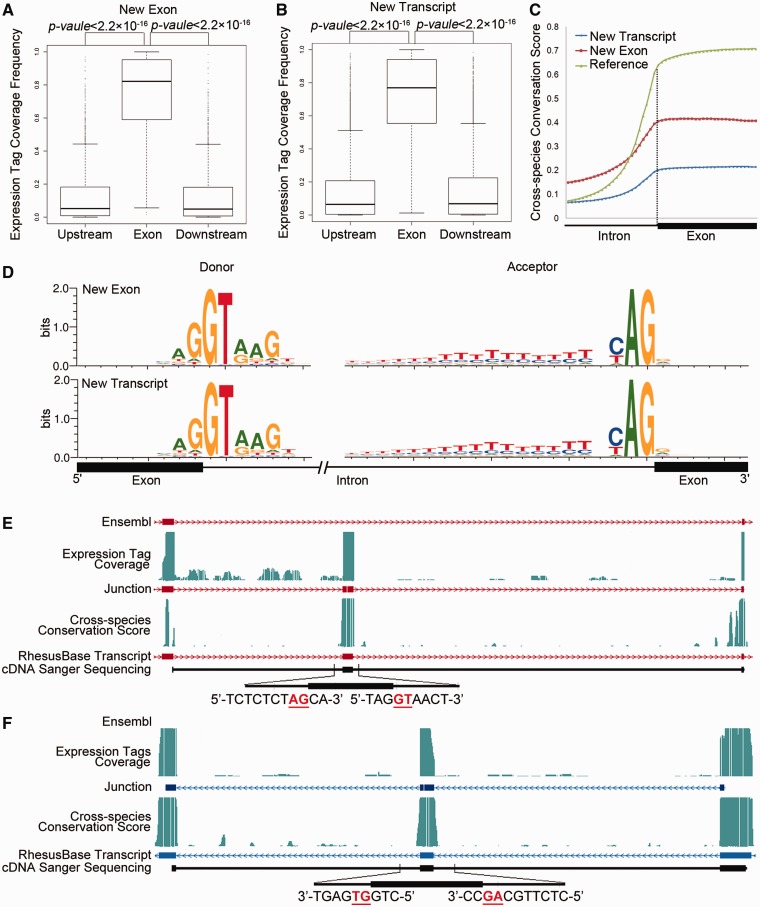
Evaluation of new exons and transcripts absent in Ensembl annotation. (**A, B**) Normalized mRNA-Seq expression tag coverage in exonic regions, upstream and downstream intronic regions, for revisions adding missed exons (A) or transcripts (B). (**C**) Intron–exon distributions of cross-species conservation score. Reference: exons in rhesus macaque supported by both gene models; New Exon: missed exons on the basis of Ensembl annotation; New Transcript: exons in new transcripts identified in this study. (**D**) Sequence motifs flanking the splicing junctions for new exons and transcripts. Distributions were calculated using 2 427 new exons (New Exons) and 24 295 exons in 8057 new transcripts (New transcripts). (**E** and **F**) Two examples are shown for the fine-scale structure of new exons missed by Ensembl (E) and new transcripts (F). Both the previous gene models (Ensembl) and the revised gene models (RhesusBase) are shown. RNA-Seq expression tag coverage, splicing junctions, cross-species conservation score, and sequenced cDNA fragments were aligned accordingly. Sequences surrounding the splicing junctions are also illustrated, in which GT-AG sites are highlighted in red.

Overall, we evaluated the three types (Figures 1–3) of refinements of transcript structures from the perspective of the distributions of the RNA-Seq expression tags (Figures 1A, 3A and B), distributions of the cross-species conservation scores (Figures 1B and 3C), as well as the sequence motif flanking the splicing junctions (Figures 1C and 3D) and the 5′- or 3′-end of the revised transcripts (Figures 2A–C). First, a series of Perl (v5.12.2) scripts were implemented to evaluate and visualize the distributions of the RNA-Seq expression tags. Then, we calculated cross-species conservation scores according to the previously reported pipeline (Supplementary Figure S4) (7). Finally, we calculated and visualized the sequence motifs flanking the donor/acceptor splice sites using WebLogo (v3.2). The ChIP-Seq dataset on histone H3 lysine 4 trimethylation (H3K4me3) was downloaded and processed on the basis of the previously reported pipeline in the original papers, which was further used to evaluate the completeness of 5′-UTRs. All statistical analyses were performed using R packages (v2.13.1).

### RNA isolation, cDNA synthesis, PCR and Sanger sequencing

The monkey tissue samples used were obtained from the Institute of Molecular Medicine in Peking University. RNA isolation, cDNA synthesis and sequencing were performed as described previously (25), using glyceraldehyde-3-phosphate dehydrogenase (Applied Biosystems) as an endogenous control. The PCR primers used in this study are listed in Supplementary Table S2.

### Integration of functional genomics data in rhesus macaque

**Figure 4.**
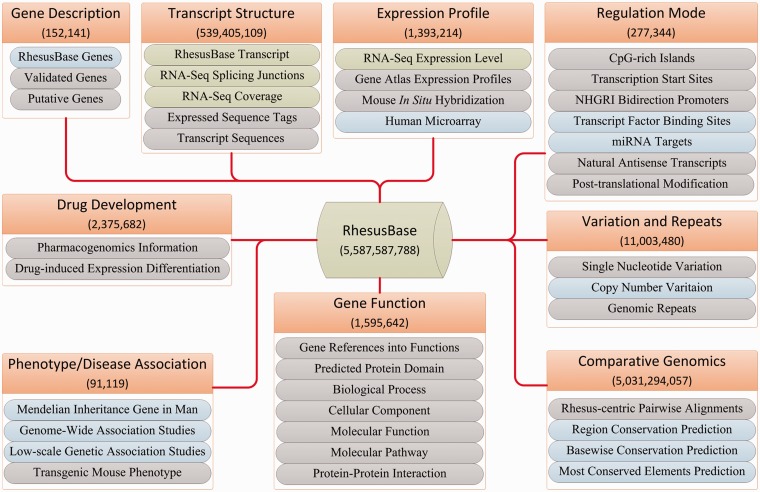
RhesusBase data integration and abstraction. Nine functional categories of annotation were integrated and standardized: Gene Description, Gene/Transcript Structure, Expression Profile, Regulation Mode, Variation and Repeats, Comparative Genomics, Gene Function, Phenotype/Disease Association and Drug Development. Detailed descriptions of annotations in each functional category are illustrated. Annotations integrated from in-house datasets are shown in green boxes, those processed from public databases in blue boxes and those extracted directly from public databases in grey boxes. The total numbers of entries in each functional category are shown.

First, in-house functional genomics data in the rhesus macaque were processed and integrated according to standardized pipelines (26) (Figure 4). Second, through the PUBMED keywords query ‘(genome OR transcriptome OR proteome) AND (rhesus macaque)’, we accessed public functional genomics studies and re-analysed the raw data according to the pipelines reported in the original studies. We designed standardized criteria for meta-data extraction and storage (Supplementary Table S3). Detailed information such as sample information, types of experimental platforms and treatments, literature information, and genotype-phenotype correlation information were carefully curated and integrated (Figure 4 and Supplementary Table S3). Third, a series of bash and Perl scripts were implemented to download, manage and process the data from >60 currently available databases (Figure 4 and Table 3). For each site in the monkey genome, cross-species conservation score was also calculated and integrated (Supplementary Methods and Supplementary Figure S4). LiftOver (7) was introduced for data transformation and standardization. Overall, functional annotations from >60 categories of public and in-house resources were integrated, with >5 billion annotation entries (Figure 4 and Table 3).

**Table 3.** Statistics for RhesusBase functional genomics annotations

Categories	Resources	All entries (Rhesus)	All gene coverage (Rhesus)	References
Gene description				
RhesusBase genes	This study	22 283^a^ (18 406^b^)	22 283^c^ (18 406^d^)	(7,34)
Validated genes	RefSeq	2 588 (2 588)	2 541 (2 541)	(35)
Putative genes	Ensembl, N-SCAN, SGP, Geneid, miRBase, GtRNAdb	127 271 (127 271)	31 416 (31 416)	(7,31,35–40)
Transcript structure				
RhesusBase transcripts	This Study, Public Data	50 847 (50 847)	28 634 (28 634)	This study
RNA-Seq coverage	This Study, Public Data	537 867 932 (537 867 932)	16 462 (16 462)	This study, (5,13–17)
Splicing junctions	This Study, Public Data	1 380 988 (1 380 988)	16 992 (16 992)	This study, (5,13–17)
Expressed sequence tags	GenBank, dbEST, UCSC	72 657 (72 657)	8 832 (8 832)	(6,7,41)
Transcript sequences	RefSeq	32 685 (32 685)	17 575 (17 575)	(35)
Expression profile				
RNA expression identified by RNA-Seq	This Study, Public Data	1 332 656 (982 226)	22 198 (16 809)	This study, (5,13–17,32)
RNA expression identified by *in situ* hybridization	Alan Brain Atlas	12 397 (0)	9 218 (0)	(42)
RNA expression identified by cDNA microarray	BioGPS, Alan Brain Atlas	48 161 (0)	20 795 (0)	(42,43)
Regulation Mode				
Transcriptional regulation	UCSC, Public Data	235 086 (235 086)	11 601 (0)	(7,15,44,45)
Posttranscriptional regulation	This Study, Argonaute, TarBase, PicTar, TargetScan, miRanda	82 355 (82 355)	1 625 (1 520)	(46–51)
Natural-antisense regulation	NATsDB, TransMap	37 868 (0)	5 463 (5 463)	(52,53)
Posttranslational modification	dbPTM	4 390 (4 390)	223 (0)	(54)
Variation and repeats				
Single nucleotide variation	This Study, dbSNP, CMSNP, MamuSNP, MonkeySNP	5 682 738 (5 500 294)	17 430 (15 743)	(9–11,55)
Copy number variation	dbVar, DGV	29 593 (337)	6 068 (104)	(8,56)
Genomic repeats	UCSC	5 291 149 (5 291 149)	15 445 (15 445)	(7,57)
Comparative genomics				
Rhesus-centric pairwise alignments	UCSC	32 487 843 (32 487 843)	17 603 (17 603)	(7)
Cross-species conservation score prediction	UCSC	4 998 806 214 (4 998 806 214)	16 435 (16 435)	This study, (7)
Gene function				
Related publication	NCBI	544 499 (269)	171 (171)	(34)
Predicted protein domain	InterPro	28 517 (28 517)	8 399 (8 399)	(58)
Biological process, cellular component and molecular function	Gene Ontology	191 251 (0)	11 850 (0)	(59)
Molecular pathway	KEGG, Reactome, BioCarta, PID	12 346 (187)	4 106 (4 106)	(60–62)
Protein–Protein Interaction	IntAct, HPRD, DIP, BioGRID, BioCyc, STRING	819 029 (672 864)	10 606 (10 606)	(27,63–67)
Phenotype and disease association				
Human inheritance disease	OMIM	9 935 (0)	6 104 (0)	(68)
Genetic susceptible gene (genome-wide association study)	NHGRI Catalog of Published Genome-Wide Association Studies	4 903 (0)	3 536 (0)	(69)
Genetic susceptible gene (low-scale association study)	GAD	44 201 (0)	3 535 (0)	(70)
Transgenic mouse phenotype	MGI, PBmice	32 080 (0)	5 420 (0)	(20,71)
Drug development				
Pharmacogenomics	PharmGKB	21 072 (0)	19 495 (0)	(72)
Drug-induced differentially expressed genes	Connectivity MAP	2 354 610 (0)	9 125 (0)	(73)

^a^Total number of RhesusBase entries in rhesus macaque, human and mouse.

^b^The number of RhesusBase entries specifically for rhesus macaque.

^c^The number of monkey genes with RhesusBase annotations from rhesus macaque, human and mouse.

^d^The number of genes with RhesusBase annotations specifically from rhesus macaque.

### Development of RhesusBase management system and interactive user interfaces

**Figure 5.**
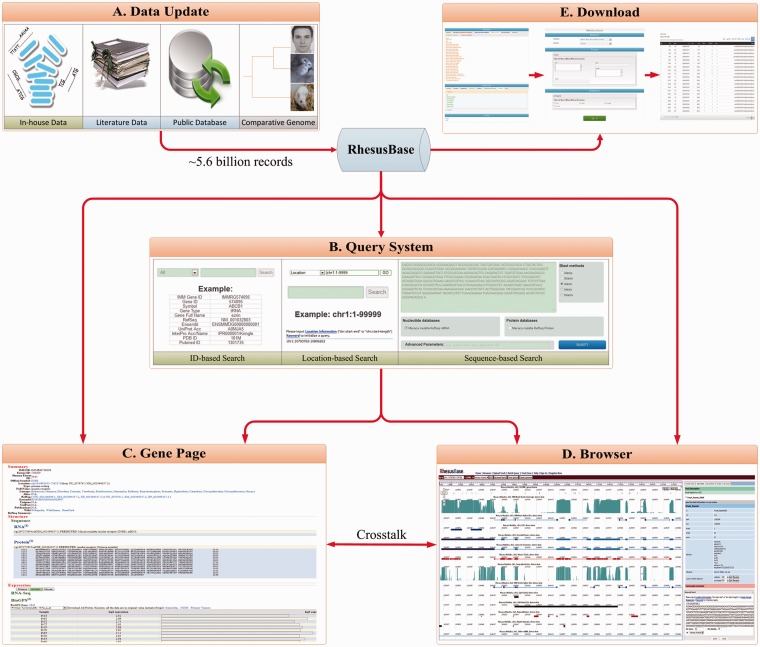
Overview of RhesusBase management system and interactive user interfaces. A comprehensive database management system and five highly interactive user interfaces were developed to support data storage, updating (**A**), retrieval (**B**), display (**C**, **D**) and downloading (**E**) in RhesusBase. A database update module was developed to facilitate the efficient updating of RhesusBase as more public or in-house functional data become available (A). Keywords, location and sequence-based query systems were developed to facilitate the retrieve of functional annotations from RhesusBase (B). Through this information retrieval system, users are referred to two different view modes to display the annotations, that of a gene-centric view (C) and a position-centric browser view (D). A Biomart-based download system was also developed for the offline use of RhesusBase annotations (E).

We developed a database, the RhesusBase, with MySQL relational schema to manage the meta-data. We also implemented highly interactive user interfaces to support the data storage, update, display, retrieve and download of the function annotations (Figure 5), using various web development technologies such as HTML, CSS, JavaScript (jQuery), AJAX (EXTJS), Java and JSP. Apache was used as the web server, with Tomcat as the JSP parser. A genome browser was developed on the basis of ABrowse (28). A Biomart-based download system (29) was also developed to facilitate the offline use of RhesusBase annotations. All annotations and database schema in RhesusBase are freely accessible at http://www.rhesusbase.org.

## RESULTS

### Correction of gene models in 28.7% Ensembl macaque transcripts

As noted earlier, for the majority of genes in the rhesus macaque, the transcript structures were putatively inferred due to scarce monkey mRNA and EST data. Recently developed deep sequencing technology made it possible to quickly generate expression tags in the rhesus macaque, whereas even when using RNA-Seq technology by selecting uniquely mapped expression tags on the genome (12), gene boundaries are difficult to determine considering the widely distributed *cis*-natural antisense events in primates (30). We thus performed a strand-specific RNA-Seq study in 10 rhesus monkey tissues from one individual to identify polyadenylated mRNAs. More than 1.2 billion 90-bp paired-end expression tags were generated and sequenced with high quality, in which 876 million tags were mapped uniquely to the rhesus monkey genome. Detailed descriptions of the data collection, expression tags mapping and RNA-Seq quality control were presented in (26).

Using the rhesus macaque genome and transcriptome annotations of Ensembl (31) as references, we assembled the fine-scale transcript structures on the basis of the distribution of mRNA expression tags on the genome, as well as the splicing sites indicated by expression tags across splicing junctions (Materials and Methods). Briefly, the expression of 351 311 (97.4%) putatively annotated exons by Ensembl were verified by RNA-Seq expression tags. In addition, 273 967 splicing junctions (86.2%) were covered by at least one RNA-Seq fragment (Table 1), supporting 250 733 (78.9%) Ensembl-annotated exon borders. These statistics indicate that the coverage of the RNA-Seq data we generated was deep enough to accurately evaluate the fine-scale transcript structure. In addition, the putative transcript structures by Ensembl annotation are largely convincing, partly due to the highly conserved transcript structures between rhesus macaque and other well-annotated genomes such as human and mouse.

However, we found that the fine-scale transcript structures in at least 28.7% of the Ensembl macaque transcripts were partially mis-annotated, mainly in three ways: mis-annotated exon/intron boundaries, incomplete 5′- or 3′-UTRs and missed constitutive exons or transcripts (Table 2). First, although most of the splicing junctions were verified, 4054 junctions in 2947 transcripts (6.9%) were mis-annotated, supported by at least two independent expression tags across the splicing junction (Table 2 and Supplementary Dataset S1). A total of 3 401 events occurred between coding exons, 1292 in which a frame-shift was introduced (Supplementary Dataset S1). Second, 5′- or 3′-UTRs in 2267 or 7917 transcripts (5.3 or 18.5%) were extended on the basis of the mRNA fragment distribution across the genome (Table 2 and Supplementary Datasets S2 and S3). Third, 2427 new exons were identified in 1602 transcripts (3.7%), supported by convincing mRNA fragment clusters, which were further connected to known gene model by RNA-Seq expression tags across splicing junctions (Table 2 and Supplementary Dataset S4). Finally, we also identified 8057 new transcripts in the rhesus macaque genome. On the basis of the current gene annotation in rhesus macaque, these transcripts were located in intergenic regions, while the RNA-Seq data suggested convincing expression of these transcripts (Materials and Methods and Supplementary Dataset S5).

We refined 16 665 events in 12 303 Ensembl transcripts across the rhesus macaque genome. If looser criteria were used in processing RNA-Seq data, as many as 16 587 Ensembl transcripts (38.7%) were modified (Supplementary Methods and Supplementary Table S1). These revisions would contribute significantly to biochemical, molecular biological and genetics studies in the monkey research community.

### The transcript structures in rhesus macaque were convincingly refined

We evaluated the three types of refinements on transcript structures in the rhesus macaque, as well as new transcripts identified. First, we evaluated the 4054 refined exon/intron boundaries from the perspective of the exon–intron distributions of the RNA-Seq expression tags, distributions of the cross-species conservation scores, and the sequence motif flanking the splicing sites. In a typical mRNA-Seq assay, the distribution of expression tags should highly enrich in exonic compared with intronic regions (32). In addition, the cross-species conservation scores in exonic regions should be higher than in intronic regions due to purifying selection (7). As expected, the coverage of expression tags in exon regions was markedly higher than that in intronic regions (Figure 1A, Mann–Whitney test, *P* value < 2.2e−16) on the basis of the revised gene models, instead of the previous models (Figure 1A). In addition, the distribution of cross-species conservation score between exons and introns were consistent with new gene models (Figure 1B, Mann–Whitney test, *P* value < 2.2e−16), instead of the previous one (Figure 1B). Especially, compared with the previous gene models, clear sequence motifs were detected flanking the revised splicing junctions (Figure 1C), consistent with the motifs generated by well-accepted splicing sites in rhesus macaque as positive controls (Figure 1C), or those reported in previous studies in human (33). These items of evidence suggest that the refinements on exon/intron boundaries are largely convincing. One example of a revised transcript is shown, validated experimentally by mRNA reverse transcription polymerase chain reaction followed by cDNA sequencing (Figure 1D and Supplementary Table S2).

Among the 4054 events for exon/intron boundary revision, 1292 occurred between coding exons and introduced frame-shift on previously annotated open reading frames. Surprisingly, on the basis of the previous gene models, most of these transcripts had intact open reading frames, encoding proteins with clear homology in human (BLASTP *E* value < 10e−5). Strikingly, in 1095 (84.8%) of these events, another nearby unusual annotation, such as putative indel, putative exon and mis-annotated exon boundary, was detected on the basis of Ensembl annotation (Supplementary Figures S1 and S2). These unusual annotations are unlikely to be true from the perspective of the RNA-Seq expression tags distribution, cross-species conservation score distribution, and the sequence motif flanking the splicing sites (Supplementary Figure S1). These double mistakes on the transcript structure rescued the open reading frames and created largely intact ORFs by current Ensembl annotations (Supplementary Figures S1 and S2). For these transcripts annotated with double mistakes by Ensembl, the revised gene models were experimentally verified in four randomly selected cases (Supplementary Figure S2 and Supplementary Table S2). This systematic error in automatic gene structure annotation is cautious, especially in genetics studies using rhesus macaque as model animals.

Then, for the 10 184 events to extend 5′- or 3′-UTRs of transcripts, the exon–intron distribution patterns of both the RNA-Seq expression tags and cross-species conservation score support the modified gene models (Supplementary Figure S3, Mann–Whitney test, *P* value < 2.2e−16). Especially, an enriched AAUAAA hexamer of the poly(A) signal was detected near the end of the revised 3′-UTRs, compared with negative controls generated using flanking regions near the start site of these transcripts (Figure 2A). Weaker enrichment of AAUAAA was detected based on the previous gene model, indicating the combination of mis-annotated transcript structure and alternative 3′-UTR splicing on these transcripts by the current annotations (Figure 2A). Actually, these mis-annotated transcript structures partly contributed to the genome-wide shift of AAUAAA distribution in rhesus macaque to the downstream region of the transcript, compared with the human genome (Figure 2B). To evaluate the completeness of 5′-UTRs, we further integrated a recent ChIP-Seq dataset to identify histone H3 lysine 4 trimethylation (H3K4me3) sites in rhesus macaque (15), indicators of transcription start sites. For the transcripts with revised 5′-UTRs, the distribution of the H3K4me3 sites around the previously defined transcription start sites differed from the reference, calculated using genes with un-modified gene models, while the distribution using the refined gene models was consistent (Figure 2C). The gene structures in two revised transcripts (one for 3′-UTR and another for 5′-UTR revision) are shown, experimentally verified by Sanger sequencing of cDNAs extracted from the corresponding monkey tissues (Figure 2D and E and Supplementary Table S2).

Similar evaluations were performed on 2427 new exons as well as 8057 brand-new transcripts absent from the current Ensembl annotation (Figure 3). Both the new exons and transcripts were convincing from the perspective of RNA-Seq expression tag coverage (Figure 3A and B), cross-species conservation score (Figure 3C) and sequence motifs near the splicing junctions (Figure 3D), indicating accurate refinements on the gene structures. Two experimentally verified genes, one for a transcript with a missed exon and another for a brand-new transcript, are shown as demonstration cases for this type of revision (Figure 3E and F and Supplementary Table S2).

Overall, the fine-scale transcript structures in at least 28.7% of the monkey Ensembl transcripts were convincingly refined in this study, posing a good supplement to the current Ensembl annotations on gene and transcript structures in the rhesus macaque.

### Comprehensive integration of functional genomics data in rhesus macaque

In the framework of well-defined gene structures, we further integrated in-house generated functional genomics data, as well as public available data scattered in the literature and specialized databases, to develop a well-annotated genomic context in the rhesus macaque (Figure 4 and Table 3). Briefly, three types of data resources were considered and integrated: First of all, as a primate center with international AAALAC standards, we generated masses of functional genomics data in the rhesus macaque especially using the deep sequencing technology. These in-house data were processed and integrated with standardized pipelines (Figure 4 and Table 3). Second, through the PUBMED keywords query, we accessed all functional genomics studies in the rhesus macaque, such as high-throughput annotations on gene expression profiles, transcription factors and microRNA binding sites generated by deep sequencing-based RNA-Seq, ChIP-Seq and CLIP-Seq technology. We re-analysed the raw data and designed standardized criteria for meta-data extraction and storage. Detailed meta-data such as sample information, types of experimental platforms and treatments, literature information and genotype-phenotype correlation information were carefully curated and integrated (Figure 4 and Table 3). Third, information in >60 currently available databases was curated and integrated to annotate the rhesus macaque genome from multiple perspectives (Table 3). Overall, for each gene in the rhesus macaque, functional annotations were integrated from nine functional categories: gene descriptions, genetic variations and repeats, gene and transcript structure, regulation mode, expression profile, gene function (including biological processes and pathways), and comparative genomics as well as disease association and drug development (Table 3 and Figure 4).

To maximize the utility of the functional annotation system, for each gene in the rhesus macaque, we also integrated all related annotations in human and mouse, as references to fully understand the monkey genome (Table 3 and Figure 4). In addition, for each site in the monkey genome, we calculated cross-species conservation scores to facilitate rhesus macaque-centric comparative genomics studies (Figures 4 and 5A). Overall, functional annotations from >60 categories of public and in-house resources were integrated, with >5 billion annotation entries (Figure 4).

### RhesusBase: a ‘one-stop’ resource for the monkey research community

We developed RhesusBase with a comprehensive database management system and highly interactive user interfaces, to support the data storage, update, display, retrieval and download of the described functional annotations in the rhesus macaque (Figure 5). First, keywords, location and sequence-based query systems were developed to facilitate the retrieve of functional annotations in RhesusBase (Figure 5B). Through this user-friendly information retrieval system, users are referred to two different view modes for the annotations, a gene-centric view and a position-centric browser view, depending on their retrieval options. In the gene-centric view (Figure 5C), each gene in the rhesus macaque was assigned one page, in which detailed annotations were arranged and visualized in different functional categories, such as genes and transcript structure, expression, regulation, variation and repeats, phenotypes and disease, function, drug design and comparative genomics (Figure 5C). For each gene, functional annotations in human and mouse orthologs were also integrated to facilitate functional studies in the rhesus macaque. In position-centric view (Figure 5D), a genome browser was developed on the basis of ABrowse (28). More than 110 functional tracks were added onto the corresponding genomic context, illustrating refined gene and transcript structures, mRNA and EST data, RNA-Seq expression tag coverage and splicing junctions, transcription regulations, comparative genomics, variation and repeats, as well as phenotype and disease associations (Figure 5D). A Biomart-based download system (29) was also developed to facilitate the offline use of RhesusBase annotations (Figure 5E). Considering the significant role of guanosine-binding protein coupled receptor (GPCR) in drug development, we also developed an interface for 857 GPCR genes (GPCR Gateway) to facilitate the translational study of human diseases. The RhesusBase is freely accessible at http://www.rhesusbase.org, providing a ‘one-stop’ resource to facilitate molecular and translational research in the community.

## DISCUSSION

Currently, functional genomic data on the rhesus macaque are scarce. The majority of gene and transcript structures were putatively predicted on the basis of other well-annotated genomes, with only ∼1% supported by real mRNA or EST data. These *ab initio* or comparative genomics-guided predictions are largely convincing, partly due to the highly conserved transcript structures between rhesus macaque and other well-annotated genomes such as human. Actually, on the basis of the putative gene models in Ensembl (31), most transcripts encode intact open reading frames, widely used in genetics and molecular evolution studies.

Based on our strand-specific RNA-Seq data, we demonstrated that the transcript structures in 28.7% of monkey genes were partially mis-annotated. Strikingly, 1292 revisions introduced a frame-shift on previously annotated open reading frames (Figure 1). Why were these serious flaws not detectable by previous computational pipelines on the basis of *a prior* comparative genomics knowledge and why could those putative transcripts with clear frame-shift mistakes still encode intact proteins? We noted that in many cases of our revisions located on chromosome regions with atypical regulatory patterns, e.g. besides standard GT–AG splicing sites, many splicing junctions use a GC–AG splicing junction, a pattern potentially neglected by *a prior* predictors (Figure 1C). In addition, many new exons and new transcripts showed a significantly lower cross-species conservation score, another atypical pattern potentially introducing errors in computational predictions (Figure 3C). More significantly, we noted that for 84.8% of the 1292 CDS boundary revision events introducing frame-shift on a previously annotated open reading frame, another nearby mistake was detected. These double mistakes created largely intact ORF by current Ensembl annotation, a strategy to make globally optimized protein structures (Supplementary Figures S1 and S2). These predictions are largely acceptable in cases studying global patterns for monkey proteomes, but error-prone in fine-scale studies such as genetics studies, in which a single mistake on an exon–intron boundary could contribute to false-positive findings. Here, for the first time, we performed genome-wide gene structure refinement on the basis of real expression data in the rhesus macaque, which will greatly facilitate fine-scale studies in the monkey research community.

It is important to study gene functions and disease mechanisms in the framework of well-annotated genomic contexts. Although national-level annotation systems such as Ensembl for the rhesus macaque (31), UCSC Genome Browser (7) and NCBI Entrez System (34) have developed web servers to visualize monkey data, the annotations are widely scattered and putative. More recently, some monkey-oriented secondary databases have been developed, but they focus on highly specialized topics, typically for the presentation of in-house SNP data (9–11). It is also difficult for biologists to take full advantage of high-throughput data (such as RNA-Seq data). A comprehensive database of the rhesus macaque is thus urgently needed to support the monkey research community, just as ‘FlyBase’ (18), ‘WormBase’ (19), the Mouse Genome Informatics (20) do for the international fruit fly, nematode and mouse research communities. Here, we present the first comprehensive ‘RhesusBase’ effort for the monkey research community. Overall, functional annotations from >60 categories of public and in-house resources were integrated, with >5 billion annotation entries, which will substantially facilitate functional and translational studies in this field.

In a primate center built according to AAALAC standards, we have successfully developed rhesus macaque models of different complex diseases (74) and started to perform genomic biomedical studies using deep-sequencing technology (26). We will continue to update RhesusBase and release the latest annotation version every year through the web server, as more public or in-house functional data become available. RhesusBase is thus a dynamic approach to provide a ‘one-stop’ resource for the monkey research community.

## ACCESSION NUMBERS

JK840892, JK840893, JK840894, JK840895, JK840896, JK840897, JK840898, JK840899, JK840900.

## AUTHOR CONTRIBUTIONS

C.Y.L. conceived the idea. C.Y.L., R.X. and X.Z. designed the study. S.J.Z., C.J.L. and M.S. performed most of the experiments. L.K., J.Y.C., W.Z.Z., X.Z., P.Y., J.W., X.Y., N.H., Z.Y. and R.L.Z. performed part of the experiments. S.J.Z., C.J.L. and M.S. analysed the data and performed the statistical analysis. C.Y.L. wrote the manuscript. All authors read and approved the final manuscript.

## SUPPLEMENTARY DATA

Supplementary Data are available at NAR Online: Supplementary Tables 1–3, Supplementary Figures 1–4, Supplementary Methods, Supplementary Datasets 1–5 and Supplementary References [75–77].

## FUNDING

The National Natural Science Foundation of China [31171269]; the National Basic Research Program of China [2011CB518000]. The funders had no role in study design, data collection and analysis, decision to publish, or preparation of the manuscript. Funding for open access charge: The National Natural Science Foundation of China [31171269].

*Conflict of interest statement*. None declared.
